# Counting generations in birth and death processes with competing Erlang and exponential waiting times

**DOI:** 10.1038/s41598-022-14202-0

**Published:** 2022-07-04

**Authors:** Giulia Belluccini, Martín López-García, Grant Lythe, Carmen Molina-París

**Affiliations:** 1 School of Mathematics, University of Leeds, Leeds, LS2 9JT UK; 2 T-6, Theoretical Biology and Biophysics, Theoretical Division, Los Alamos National Laboratory, Los Alamos, NM USA

**Keywords:** Cell biology, Immunology, Mathematics and computing

## Abstract

Lymphocyte populations, stimulated in vitro or in vivo, grow as cells divide. Stochastic models are appropriate because some cells undergo multiple rounds of division, some die, and others of the same type in the same conditions do not divide at all. If individual cells behave independently, then each cell can be imagined as sampling from a probability density of times to division and death. The exponential density is the most mathematically and computationally convenient choice. It has the advantage of satisfying the memoryless property, consistent with a Markov process, but it overestimates the probability of short division times. With the aim of preserving the advantages of a Markovian framework while improving the representation of experimentally-observed division times, we consider a multi-stage model of cellular division and death. We use Erlang-distributed (or, more generally, phase-type distributed) times to division, and exponentially distributed times to death. We classify cells into generations, using the rule that the daughters of cells in generation *n* are in generation $n+1$. In some circumstances, our representation is equivalent to established models of lymphocyte dynamics. We find the growth rate of the cell population by calculating the proportions of cells by stage and generation. The exponent describing the late-time cell population growth, and the criterion for extinction of the population, differs from what would be expected if *N* steps with rate $\lambda$ were equivalent to a single step of rate $\lambda /N$. We link with a published experimental dataset, where cell counts were reported after T cells were transferred to lymphopenic mice, using Approximate Bayesian Computation. In the comparison, the death rate is assumed to be proportional to the generation and the Erlang time to division for generation 0 is allowed to differ from that of subsequent generations. The multi-stage representation is preferred to a simple exponential in posterior distributions, and the mean time to first division is estimated to be longer than the mean time to subsequent divisions.

## Introduction

Cells of the immune system patrol our bodies for months or years^1,2^. During an adaptive immune response, a subset of specific cells, initially a small fraction of the total population, expands as cells undergo multiple rounds of division over a few days^3^. Although most of these cells die as the infection is overcome, lasting immunity is ensured by the transformation, or “differentiation” of individual cells to a memory phenotype. The most convenient mathematical and computational models of the dynamics of cell populations, which can consider heterogeneity at the single-cell level, are Markov models. In these models, the variables describe the number of cells of each type as a function of time, and cellular events such as division, death or differentiation are defined by their associated rates; each event corresponds to a possible fate of an individual cell and cells are independent of each other. In this formulation, inter-event times are exponentially-distributed random variables, with probability density maximised at zero.

The rapid expansion of cohorts of lymphocytes is recreated in laboratories, either by stimulation in vitro or by transferring cells to lymphopenic mice. By labelling cells with carboxyfluorescein succinimidyl ester (CFSE) or cell trace violet at the beginning of an experiment, and then using flow cytometry at a later time, a cohort of cells can be classified into generations because each round of division dilutes the intracellular dye^4–9^. At the beginning of an experiment, when cells are labelled, it is considered that all cells are in generation 0. In time-lapse microscopy experiments, individual cells are tracked and correlations within family trees identified^10–17^. Over the timescales of such experiments, hours to days, it is not appropriate to treat cell division as an instantaneous event. Rather, cells are “cycling” through gap, synthesis and mitosis phases ($\hbox {G}_1$/$\hbox {G}_2$, S and M), and daughter cells cannot immediately redivide^18^. To improve on the exponential distribution, Smith and Martin proposed a model in which the time between divisions is the sum of a fixed time spent in phase B, corresponding to S/$\hbox {G}_2$/M, and a variable time spent in phase A, corresponding to $\hbox {G}_1$^19^. In the “single stochastic division” model of Hogan et al., the rate of transition from A to B phase depends on the T cell clonotype and on the number of cells competing for the same resources^20^. If there is a common molecular mechanism controlling the time spent in all phases of the cell cycle, then phase B may, instead, occupy a fixed proportion of the total time^10^. Takahashi^21,22^ divided the cell cycle into four phases, with the duration of each drawn from a Pearson type III distribution. Weber et al. postulated a delayed exponential waiting time for each of three phases, corresponding to $\hbox {G}_1$, S and $\hbox {G}_2$/M^23^.

The cyton model is a general framework for modelling proliferating lymphocytes, based on the idea that each cell has a set of competing clocks, determining its fate^13^. A number of features are incorporated in the model: generation-dependent parameters, heritable factors, and correlations between cells of the same generation^11,16,24^. When the fate of an individual cell is determined by competing internal clocks, the probability density of observed times between divisions is not the same as that of the division clock because division only happens if another fate does not. The probability density of division times is said to be “censored”^25^. When all clocks have exponential probability densities, the probability density of observed division times is also exponential due to the memoryless property of exponential random variables; this conservation of shape does not hold for non-exponential distributions, including the log-normal and Erlang distributions.

Kendall^26^ introduced the idea of cell division occurring at the end of a sequence of *k* phases, with an exponentially-distributed time spent in each phase. Luzyanina et al.^27^ made use of a similar model, where each phase identifies a cell generation, to study methodological and computational issues related to CFSE labelling data. Gamma and Erlang distributions have been considered to model a cell’s time to division^28,29^. Yates et al.^30^ incorporated the idea of a sequence of exponentially-distributed phases, also referred as *stages*, before division in a multi-stage representation of a population of dividing cells. This yields an Erlang distribution of times to division, while retaining some of the mathematical and computational advantages of the exponential distribution^30^. The number of stages and their mean duration can be used as free parameters to compare with experimental data^31,32^. On the other hand, the internal stages are a mathematical construct that do not directly correspond to biological phases.

In this manuscript we adopt a multi-stage representation of the cell cycle and we include cell death as a competing fate. In the simplest case, obtained by assuming identical birth and death rates across stages, we derive an analytical expression for the expected number of cells in each stage as a function of time, and study the limiting behaviour of the system as $t \rightarrow + \infty$. We also consider cell generations, in order to make theoretical predictions comparable to CFSE experimental data^20^. The expected number of cells in each generation is found in the case of constant birth and death rates, and number of stages across generations. Although arising from different motivations, our multi-stage model with cell generations can be accommodated within the cyton framework, with the progressor fraction equal to one, division and death clocks following Erlang and exponential distributions, respectively. We show the applicability of this stochastic approach by calibrating our multi-stage model with cell generations and its exponential version with CFSE data from two populations of murine T cells^20^. Model calibration is performed making use of Approximate Bayesian Computation Sequential Monte Carlo (ABC-SMC) approaches^33^.

In the “Methods: multi-stage models of cell division and death with a Markovian framework” section, we introduce the multi-stage model including cell death and obtain analytical results for the multi-stage framework under some conditions on the model parameters. In the “Case study: lymphopenia-induced proliferation” section, we calibrate the multi-stage model with cell generations by making use of CFSE data from Hogan et al.^20^. Its performance is compared with a simple exponential model of cell division. A final discussion is provided in the “Discussion” section.

## Methods: multi-stage models of cell division and death with a Markovian framework

We present a multi-stage (MS) model of the time between cell divisions. Cells pass through a sequence of *N* stages before dividing. The stages are not directly related to the biological phases of the cellular cycle. The time to progress from stage *j* to the next one, $j+1$, is an exponentially-distributed random variable with mean $1/\lambda ^{(j)}$. We will refer to these rates, $\lambda ^{(j)}$, $j=1,\ldots ,N$, as *birth* rates. Times to death are also distributed exponentially, with per cell *death* rate $\mu$. Thus, at each stage, each cell may either proceed to the next one, with probability $\lambda ^{(j)}/(\lambda ^{(j)}+\mu )$, or die, with probability $\mu /(\lambda ^{(j)}+\mu )$. The time increment is a random variable, following the exponential distribution with mean $1/(\lambda ^{(j)}+\mu )$.

**Figure 1:**
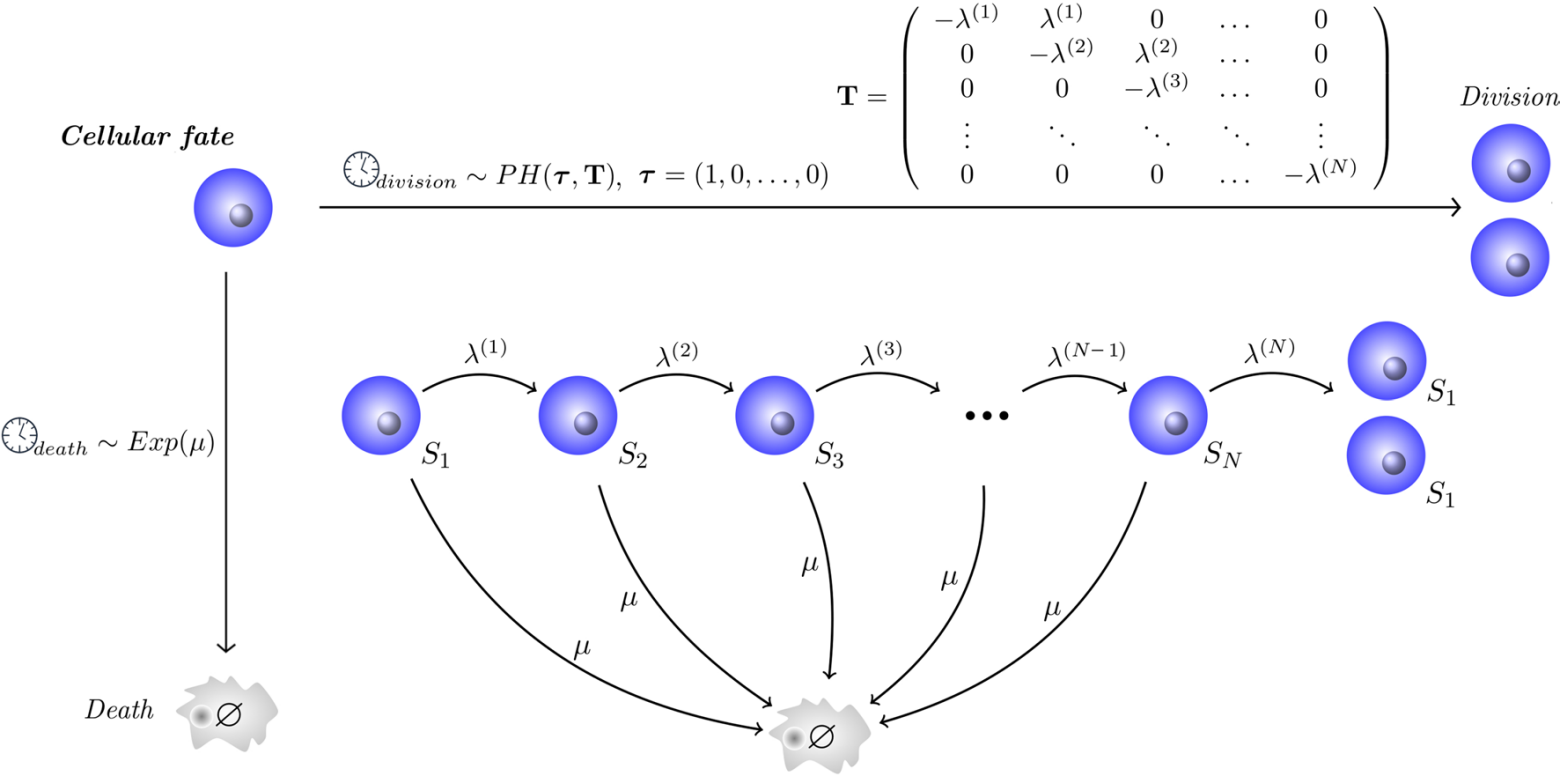
Multi-stage model of cell division and death (MS model). The cell cycle is divided into *N* different stages. A cell has to visit *N* stages in order to divide. At each stage *j*, $j=1, \ldots ,N$, the cell may proceed to the next stage, with birth rate $\lambda ^{(j)}$, or die, with death rate $\mu$.

Figure 1 illustrates the dynamics. Our multi-stage model is equivalent to considering two independent clocks for cell division and death, which compete to decide the cellular fate. The time-to-death clock follows an exponential distribution with rate $\mu$, while the division time follows a continuous phase-type distribution with parameters $\varvec{\tau }$ and $\mathbf{T}$^34^. A particular choice of phase-type distribution is the $Erlang(\lambda ,N)$, which is a concatenation of *N* identically distributed exponential steps, where all birth rates are equal: $\lambda ^{(j)}=\lambda$, $j=1,\ldots ,N$. The case $\mu =0$ has been considered by Yates et al.^30^.

The number of cells in stage *j* at time *t*, is the random variable $S_j(t)$, $j=1,\ldots ,N$. Let $M_j(t)={\mathbb {E}}[S_j(t)]$, be the expected value of $S_j(t)$. The following set of differential equations may be obtained by considering the events that can happen in a short time interval:

2.1 $$\begin{aligned} \frac{\mathrm{d}M_j(t)}{\mathrm{d}t}= {\left\{ \begin{array}{ll} 2 \lambda ^{(N)} M_N(t) -(\lambda ^{(1)}+\mu ) M_1(t), &{} \text {if} \ j=1, \\ \lambda ^{(j-1)} M_{j-1}(t) - (\lambda ^{(j)} + \mu ) M_j(t), &{} \text {if} \ j=2, \ldots , N. \end{array}\right. } \end{aligned}$$

When we extend the MS model to assign a generation to each cell, we refer to the model as the MS-G model. In this way, mean quantities can be compared with CFSE experimental data^8^. Histograms of CFSE intensity display a series of peaks, each corresponding to a *generation*, or a number of divisions over the course of the experiment^35^.

**Figure 2:**
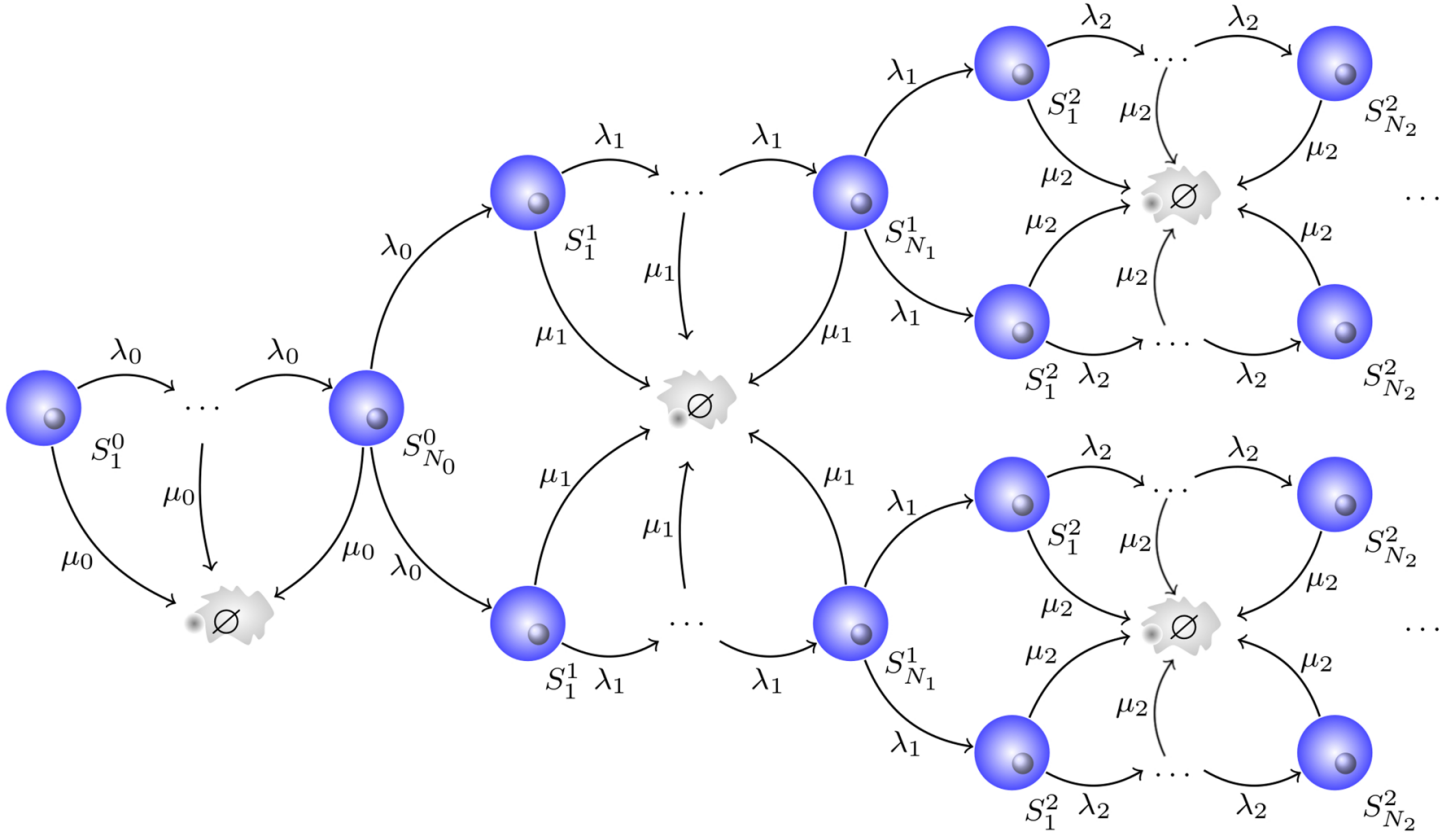
Multi-stage model with cell generations (MS-G model). Each cell in the first stage of generation 0 has to visit all the $N_0$ compartments (or stages) in order to divide. When cells arrive at the last stage of generation 0, $N_0$, they may divide with birth rate $\lambda _0$, or die with death rate $\mu _0$. If a cell divides, its daughter cells join the first compartment of the next generation, and the process continues.

In the MS-G model, generation $g\ge 0$ is split into $N_g$ different stages. The notation $N_g$ reflects the fact that the number of stages may depend on the generation *g*. A cell in generation *g* has to sequentially visit all $N_g$ compartments to divide. On the other hand, cells might also die at any stage of the cycle. As depicted in Fig. 2, if a cell belongs to generation *g* and is in compartment *j*, $j=1,\ldots ,N_g-1$, it may proceed to the following stage, with birth rate $\lambda _g$, or die with death rate $\mu _g$. Again the notation reflects the potential for these rates to depend on the generation. When a cell reaches the last stage, $N_g$, of generation *g* and divides, its two daughters will join the first compartment of generation $g+1$. In summary, given a cell in generation *g*, its time to division follows an Erlang distribution with parameters $(\lambda _g,N_g)$, whereas its time to death follows an exponential distribution with rate $\mu _g$. These distributions correspond to two independent competing clocks to control cellular fate, similarly to those considered in Fig. 1.

The number of cells in stage *j* of generation *g* at time *t* is the random variable $S^g_j(t)$, $g \ge 0$, $j=1,\ldots ,N_g$. Let $M_j^g (t)={\mathbb {E}}[S_j^g (t)]$ be the expected value of $S_j^g (t)$. The following set of differential equations may be obtained by considering the events that can happen in a short time interval:

2.2 $$\begin{aligned} \frac{\mathrm{d}M^g_j(t)}{\mathrm{d}t}= {\left\{ \begin{array}{ll} -(\lambda _0 + \mu _0) M^0_1(t), &{} \text {if} \ g=0, \ j=1, \\ \lambda _g M^g_{j-1}(t) - (\lambda _g + \mu _g) M^g_j(t), &{} \text {if} \ g \ge 0, \ j=2, \ldots , N_g, \\ 2 \lambda _{g-1} M^{g-1}_{N_{g-1}}(t) - (\lambda _g + \mu _g) M^g_1(t), &{} \text {if} \ g \ge 1, \ j=1. \end{array}\right. } \end{aligned}$$

We are interested in computing the mean number of cells over time for the MS and MS-G models. Specifically the MS-G model will provide the mean number of cells in each generation, and thus, can be used together with CFSE data to obtain division and death rates. When division times are Erlang distributed (MS model), or if one considers that those Erlang distributions are identical across generations (MS-G model), it is possible to carry out a comprehensive analytical study. This is shown in the “Analytical results” section.

When convenient analytical solutions cannot be obtained, (2.1)–(2.2) can be solved numerically in different ways. For example, for the MS-G model, and keeping in mind our interest in modelling CFSE data, we assume there exists a maximum generation *G* that can be measured by the dye. Thus, one might be interested in following cells within generations $g=0,\ldots ,G$. For these generations, Eq. (2.2) can be solved by making use of the matrix exponential. To this end, let ${\varvec{M}}(t)$ be the column vector of the mean number of cells in each stage and generation as time evolves, i.e.,

$$\begin{aligned} {\varvec{M}}(t)&= \left( M^0_1(t), \dots , M^0_{N_0}(t), M^1_1(t), \dots , M^1_{N_1}(t), \dots , M^G_1(t), \dots , M^G_{N_G}(t) \right) ^T\\ &\quad = (\mathbf{M}_0(t)^T,\mathbf{M}_1(t)^T,\dots ,\mathbf{M}_G(t)^T)^T, \end{aligned}$$

which has length $\sum _{g=0}^G N_g$, and where the column sub-vectors $\mathbf{M}_g(t)$ contain the mean number of cells across stages in generations $g=0,\ldots ,G$. Let us also define the coefficient matrix

$$\begin{aligned} \mathbf{A}&= \left( \begin{array}{cccccc} \mathbf{A}_{00} &{} \mathbf{0}_{N_0\times N_1} &{} \mathbf{0}_{N_0\times N_2} &{} \cdots &{} \mathbf{0}_{N_0\times N_{G-1}} &{} \mathbf{0}_{N_0\times N_{G}}\\ \mathbf{A}_{10} &{} \mathbf{A}_{11} &{} \mathbf{0}_{N_1\times N_2} &{} \cdots &{} \mathbf{0}_{N_1\times N_{G-1}} &{} \mathbf{0}_{N_1\times N_{G}}\\ \vdots &{} \ddots &{} \ddots &{} \ddots &{} \vdots &{} \vdots \\ \mathbf{0}_{N_{G-1}\times N_0} &{} \mathbf{0}_{N_{G-1}\times N_1} &{} \mathbf{0}_{N_{G-1}\times N_2} &{} \cdots &{} \mathbf{A}_{G-1,G-1} &{} \mathbf{0}_{N_{G-1}\times N_{G}}\\ \mathbf{0}_{N_{G}\times N_0} &{} \mathbf{0}_{N_{G}\times N_1} &{} \mathbf{0}_{N_{G}\times N_2} &{} \cdots &{} \mathbf{A}_{G,G-1} &{} \mathbf{A}_{G,G}\\ \end{array}\right) , \end{aligned}$$

where

$$\begin{aligned} \mathbf{A}_{gg}&= \left( \begin{array}{cccccc} -(\lambda _g+\mu _g) &{} 0 &{} 0 &{} 0 &{} \cdots &{} 0 \\ \lambda _g &{} -(\lambda _g+\mu _g) &{} 0 &{} 0 &{} \cdots &{} 0 \\ 0 &{} \lambda _g &{} -(\lambda _g+\mu _g) &{} 0 &{} \cdots &{} 0 \\ \vdots &{} \ddots &{} \ddots &{} \ddots &{} \ddots &{} \vdots \\ 0 &{} \cdots &{} 0 &{} \lambda _g &{} -(\lambda _g+\mu _g) &{} 0\\ 0 &{} \cdots &{} 0 &{} 0 &{} \lambda _g &{} -(\lambda _g+\mu _g) \\ \end{array}\right) ,\\ \mathbf{A}_{g,g-1}&= \left( \begin{array}{cccc} 0 &{} \cdots &{} 0 &{} 2 \lambda _{g-1}\\ 0 &{} \cdots &{} 0 &{} 0\\ \vdots &{} 
\ddots &{} \vdots &{} \vdots \\ 0 &{} \cdots &{} 0 &{} 0 \\ \end{array}\right) . \end{aligned}$$

$\mathbf{A}_{gg}$ is a square $N_g \times N_g$ matrix, whereas $\mathbf{A}_{g,g-1}$ is a $N_g \times N_{g-1}$ matrix. ${\varvec{A}}$ is then a real square matrix of dimension $\sum _{g=0}^G N_g$, and $\mathbf{0}_{a\times b}$ represents a null matrix with dimension $a\times b$. Given the vector of the initial conditions ${\varvec{n}}_0$, which has length $\sum _{g=0}^G N_g$, the system of Eq. (2.2) can be rewritten as the following Cauchy problem

$$\begin{aligned} \left\{ \begin{array}{l} \displaystyle {\frac{\mathrm{d}{\varvec{M}}(t)}{\mathrm{d}t}} = {\varvec{A}}\cdot {\varvec{M}}(t), \\ {\varvec{M}}(0) = \varvec{n_0}. \end{array}\right. \end{aligned}$$

The solution of the system is given by ${\varvec{M}}(t) = \mathrm{e}^{{\varvec{A}} t} \varvec{n_0}$, where

$$\begin{aligned} \mathrm{e}^{{\varvec{A}} t} = \sum _{k=0}^{+ \infty } \frac{({\varvec{A}} t)^k}{k!} \end{aligned}$$

represents the matrix exponential. For efficient ways of computing this matrix, see Refs.^36–39^. Finally, we note that since CFSE data describe the number of cells in each generation, one can then compute the mean number of cells in each generation over time as

2.3 $$\begin{aligned} 
M^g(t) = \sum _{j=1}^{N_g} M^g_j(t), \qquad g \ge 0. \end{aligned}$$

Using (2.2) and (2.3), we compute the time evolution of $M^g(t)$ as follows

2.4 $$\begin{aligned} \begin{aligned} \frac{\mathrm{d}M^0(t)}{\mathrm{d}t}&= -\lambda _0 M^0_{N_0}(t) - \mu _0 M^0(t), \\ \frac{\mathrm{d}M^g(t)}{\mathrm{d}t}&= 2 \lambda _{g-1} M^{g-1}_{N_{g-1}}(t) - \lambda _g M^g_{N_g}(t) - \mu _g M^g(t), \qquad g \ge 1. \end{aligned} \end{aligned}$$

In a related approach by Zilman et al.^29^, a cell’s time to division is a gamma-distributed random variable, and time to death is exponentially distributed. Solutions are given in terms of integral equations. Here, with Erlang-distributed division times, we find a set of linear differential equations for the expected number of cells in each stage.

## Analytical results

In this Section, we show how the Markovian framework of the proposed multi-stage models provides analytical tractability under some simplifying assumptions. Our aim is to compute the mean number of cells in each stage and generation over time, especially the limiting behaviour as $t \rightarrow + \infty$.

### MS model with Erlang division time

In this Section, we consider a simple case of the MS model, where identical birth rates are assumed across different stages; that is, $\lambda ^{(j)}=\lambda ,\ j=1,\ldots ,N$. The phase-type distribution for the time to division in Fig. 1 is $Erlang(\lambda ,N)$ and the mean time to division is given by $\frac{N}{\lambda }$. Note that when $N=1$ the MS model becomes a Markov linear birth-and-death process, with birth rate, $\lambda$, and death rate, $\mu$. Equation (2.1) becomes

3.1 $$\begin{aligned} \frac{\mathrm{d}M_j(t)}{\mathrm{d}t}= {\left\{ \begin{array}{ll} 2 \lambda M_N(t) - \left( \lambda + \mu \right) M_1(t), &{} \text {if} \ j=1, \\ \lambda M_{j-1}(t) - \left( \lambda + \mu \right) M_j(t), &{} \text {if} \ j=2, \ldots , N. \end{array}\right. } \end{aligned}$$

As in Yates et al.^30^, we introduce the new variables $m_j(t) = \mathrm{e}^{(\lambda + \mu )t} M_j(t)$, $j=1,\ldots ,N$, which satisfy the following ODEs:

3.2 $$\begin{aligned} \frac{\mathrm{d}m_j(t)}{\mathrm{d}t}= {\left\{ \begin{array}{ll} 2 \lambda m_N(t), &{} \text {if} \ j=1, \\ \lambda m_{j-1}(t), &{} \text {if} \ j=2, \ldots , N. \end{array}\right. } \end{aligned}$$

We find an *N*th-order homogeneous differential equation for $m_N(t)$ that does not depend on $\mu$:

3.3 $$\begin{aligned} \frac{\mathrm{d}^N m_N(t)}{\mathrm{d}t^N} = 2 \lambda ^N m_N(t), \end{aligned}$$

together with a set of ODEs that relate $m_j(t)$ to the derivatives of $m_N(t)$ with respect to time

3.4 $$\begin{aligned} m_j(t) = \left( \frac{1}{\lambda }\right) ^{N-j} \frac{\mathrm{d}^{N-j}m_N(t)}{\mathrm{d}t^{N-j}}, \ \ j=1, \ldots , N-1. \end{aligned}$$

The solution is^30^

$$\begin{aligned} m_j(t) = 2^{1-\frac{j}{N}} \sum _{k=0}^{N-1} \; c_k \; z^{-kj} \; 
\mathrm{e}^{2^{\frac{1}{N}} \lambda z^k t}, \end{aligned}$$

$j=1,\ldots ,N$ and $t\ge 0$, where $z=\mathrm{e}^{\frac{2 \pi i}{N}}$ is the first *N*th root of unity, and $c_k$ (for $k=0, \ldots , N-1$) are constants which depend on the initial conditions.

If, at time $t=0$, there are $C_0$ cells in the first stage and zero cells in any other stage, we have

$$\begin{aligned} c_k = C_0\frac{2^{\frac{1}{N}}}{2N} \; z^k, \ \ k=0,\ldots ,N-1. \end{aligned}$$

Then the solutions of the system (3.1) in terms of the original variables are

3.5 $$\begin{aligned} M_j(t) = C_0 \; \frac{2^{\frac{1-j}{N}}}{N} \; \mathrm{e}^{-\mu t} \; \sum _{k=0}^{N-1} \; z^{(1-j)k} \mathrm{e}^{\left( 2^{\frac{1}{N}} z^k -1 \right) \lambda t},\qquad j=1,\ldots ,N. \end{aligned}$$

Therefore, the expected total number of cells in the population at time *t*, *M*(*t*), is given by

3.6 $$\begin{aligned} M(t) = \sum _{j=1}^N M_j(t) = C_0\frac{2^{\frac{1}{N}}}{2N} \mathrm{e}^{-\mu t} \sum _{k=0}^{N-1} \frac{z^k}{2^{\frac{1}{N}} z^k -1} \mathrm{e}^{\left( 2^{\frac{1}{N}} z^k -1\right) \lambda t }. \end{aligned}$$

#### Population extinction or growth

Let us consider the MS model with Erlang division time, and define $S(t)=\sum _{i=1}^NS_i(t)$, the total number of cells in the population at time $t\ge 0$, so that $M(t)={\mathbb {E}}[S(t)]$. One can define

$$\begin{aligned} p_j^{(m)} \ =\ \lim \limits _{t\rightarrow +\infty }{\mathbb {P}}(S(t)=0\ |\ S_j(0)=m,\ S_k(0)=0,\ k\ne j),\quad j=1,\dots ,N, \end{aligned}$$

the probability of population extinction at late times, for *m* cells initially in stage *j* at time $t=0$. Since cells behave independently, it is clear that $p_j^{(m)}=(p_j^{(1)})^m$, so we omit the superindex from now on and denote $p_j^{(1)}\equiv p_j$. A first-step argument leads to

$$\begin{aligned} p_i&= \frac{\mu }{\lambda +\mu }+\frac{\lambda }{\lambda +\mu } \; p_{i+1},\quad i=1,\dots ,N-1,\\ p_N&= \frac{\mu }{\lambda +\mu }+\frac{\lambda }{\lambda +\mu } \; p_{1}^2. \end{aligned}$$

These equations can be solved recursively, leading to

3.7 $$\begin{aligned} p_1&= \left\{ \begin{array}{ll} \frac{1}{(1-r)^N}-1,&{} \hbox {if }\mu <(2^{1/N}-1)\lambda ,\\ 1,&{} \hbox {if }\mu \ge (2^{1/N}-1)\lambda , \end{array} \right. \end{aligned}$$

where $r=\frac{\mu }{\mu +\lambda }$. When $N=1$, $p_1=\frac{\mu }{\lambda }$; when $N=2$, $p_1=\frac{\mu ^2+2\mu \lambda }{\lambda ^2}$.

**Figure 3:**
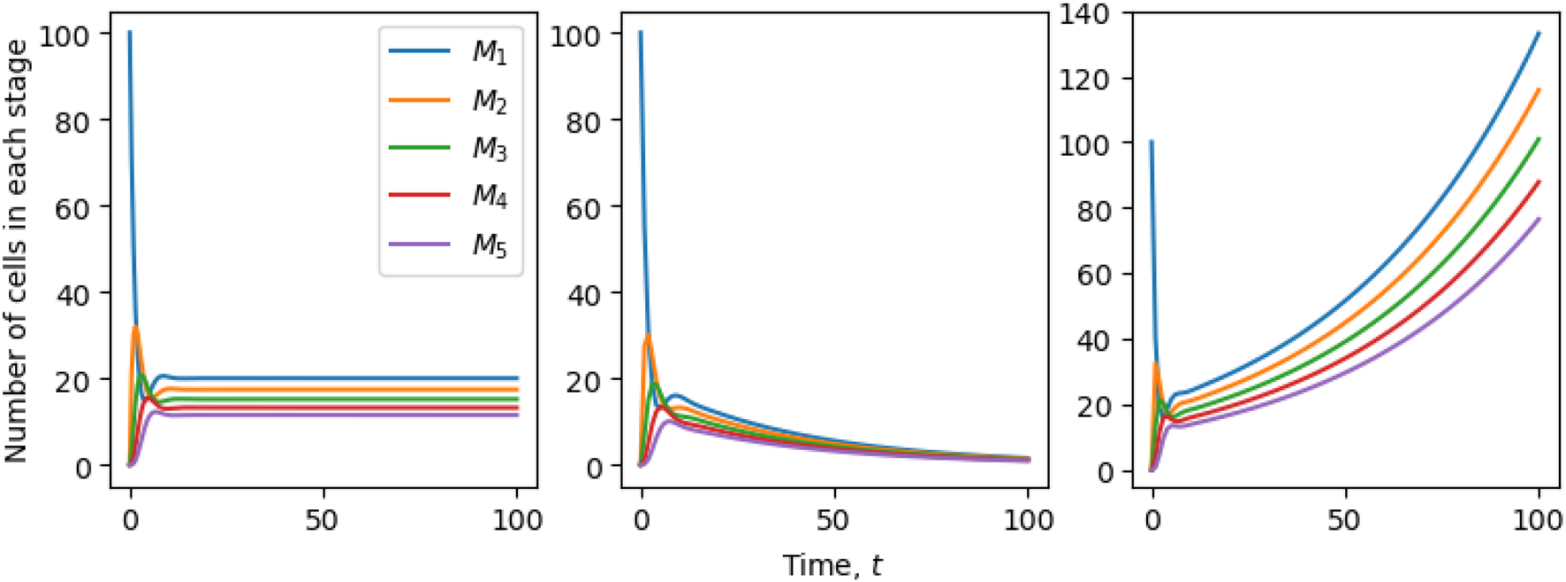
Limiting behaviour when $t \rightarrow +\infty$ of a population with an initial number of cells, $C_0 = 10^2$. Birth and death rates, $\lambda$ and $\mu$, have units of inverse time, $t^{-1}$. Left: Parameters: $N=5$, $\lambda = 0.6$, $\mu = (2^{1/N}-1)\lambda$. The population of cells in stage *j* levels out to $2^{\frac{1-j}{N}} C_0/N$ for sufficiently large times. Centre: Parameters: $N=5$, $\lambda =0.5$, $\mu = 0.1$. The population of cells at any stage becomes extinct at late times. Right: Parameters: $N=5$, $\lambda =0.8$, $\mu =0.1$. The populations grow according to (3.11) and the relation between $M_1$ and $M_5$ given by Eq. (3.9) is satisfied. For example, at $t=100$, $M_1(t) \simeq 2^{4/5}M_5(t)$.

The analytical solutions (3.5)–(3.6) provide another route to study the limiting behaviour as $t \rightarrow + \infty$. The largest term in the summation of (3.5) is the one corresponding to $k=0$. The combination $(2^{1/N}-1)\lambda -\mu$, is positive if $\mu < (2^{1/N}-1)\lambda$, negative, when $\mu > (2^{1/N}-1)\lambda$, or zero if $\mu = (2^{1/N}-1)\lambda$. Thus, extinction of the cell population is certain if $\mu > (2^{1/N}-1)\lambda$. Figure 3 (centre) shows an example of extinction when $N=5, \lambda =0.5, \mu = 0.1$ and the initial number of cells $C_0=10^2$. An example of behaviour when $\mu = (2^{1/N}-1)\lambda$ is shown in the left panel of Fig. 3.

If $\mu < (2^{1/N}-1)\lambda$ then, as $t\rightarrow +\infty$,

3.8 $$\begin{aligned} M_j(t) \simeq C_0 \; \frac{2^{\frac{1-j}{N}}}{N} \; \mathrm{e}^{-\mu t} \; \mathrm{e}^{(2^{\frac{1}{N}}-1)\lambda t}, \qquad j=1,\ldots ,N, \end{aligned}$$

and

3.9 $$\begin{aligned} \lim _{t \rightarrow +\infty } \frac{M_1(t)}{M_N(t)} = 2^{\frac{N-1}{N}}, \end{aligned}$$

which is illustrated in Fig. 3 (right). The total population size is easily obtained using

3.10 $$\begin{aligned} \frac{\mathrm{d}M(t)}{\mathrm{d}t} = \lambda M_N(t) - \mu M(t). \end{aligned}$$

As $$t \rightarrow + \infty$$,

3.11 $$\begin{aligned} M(t) \simeq C_0 \; \frac{2^{\frac{1}{N}}}{2N \left( 2^{\frac{1}{N}}-1\right) } \; \mathrm{e}^{(\sigma _N\lambda - \mu )t},\qquad \text {where } \sigma _N=(2^{\frac{1}{N}}-1)\lambda . \end{aligned}$$

**Figure 4:**
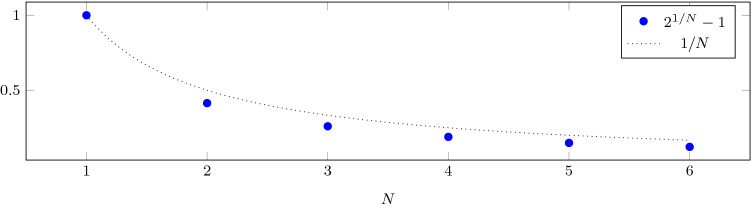
The exponent $\sigma _N$ that determines the asymptotic growth rate of the population is shown against the number of stages. The dotted line would be expected if *N* steps with rate $\lambda$ were equivalent to a single step of rate $\lambda /N$.

The exponent $\sigma _N = 2^{\frac{1}{N}} -1$, describing the late-time cell population growth, plotted in Fig. 4, is lower than would be expected if *N* steps with rate $\lambda$ were equivalent to a single step of rate $\lambda /N$. As $N\rightarrow +\infty$, we have $N\sigma _N\rightarrow \log 2$. In terms of (3.10), $\lambda \sigma _N < \lambda /N$ since $M_N(t)<1/N$ as $t\rightarrow +\infty$. Because the cell population is unevenly distributed across stages, with a bias towards earlier stages in the long run, *N* steps with rate $\lambda$ are not equivalent to a single step of rate $\lambda /N$.

#### Mean fraction of cells at each stage

We define the mean fraction of cells in each stage, $P_j(t)$, as the ratio between the mean number of cells in compartment *j* and the expected total number of cells in the population, i.e.,

3.12 $$\begin{aligned} P_j(t) = \frac{M_j(t)}{M(t)}, \ \ j=1,\ldots ,N. \end{aligned}$$

We make use of (3.1) and (3.10) to write,

3.13 $$\begin{aligned} \frac{\mathrm{d}P_j(t)}{\mathrm{d}t}= {\left\{ \begin{array}{ll} \lambda (2 P_N(t) - P_1(t) - P_1(t) P_N(t)), &{} \text {if} \ j=1, \\ \lambda (P_{j-1}(t) - P_j(t) - P_j(t) P_N(t)), &{} \text {if} \ j=2, \ldots , N, \end{array}\right. } \end{aligned}$$

which have the following steady state solution

3.14 $$\begin{aligned} P_1^*=\displaystyle {\frac{2 P_N^*}{1+P_N^*}},\quad P_j^*=\displaystyle {\frac{P_{j-1}^*}{1+P_N^*}},\ j=2, \ldots , N. \end{aligned}$$

One observes that $P_j^* < P_{j-1}^*$, $j=1,\ldots ,N-1$, which means (on average) the fraction of cells decreases stage by stage, independently of the initial distribution of cells. In fact, one can solve (3.14) to determine $P_j^*$, as follows

3.15 $$\begin{aligned} P_j^* = \left( \root N \of {2}\right) ^{N-j} \left( \root N \of {2} -1\right) , \; j=1,\ldots ,N, \end{aligned}$$

which does not depend on $\lambda$ or $\mu$. Thus, at late times the fraction of cells in each stage only depends on the number of stages considered; the parameter $\lambda$ sets the timescale of the dynamical system, and all cells are equally susceptible to death, regardless of the stage they are in.

### MS-G model with identical Erlang division times across generations

The solutions of the system (2.2) can be written in a closed analytical form in particular cases. For example, one may consider a simplified scenario where the number of stages is equal to 1 for all the generations, i.e., $N_g=1$ for all $g \ge 0$. Then, if we consider that at time $t=0$, there are $C_0$ cells in generation 0, so that $\mathbf{n}_0=(C_0,0,\ldots ,0)$, this leads to the following solutions:

3.16 $$\begin{aligned} \begin{aligned} M^0(t)&= C_0 \mathrm{e}^{-(\lambda _0 + \mu _0)t}, \\ M^g(t)&= 2^g \; C_0 \; \left( \prod _{l=0}^{g-1} \lambda _l\right) \; \sum _{i=0}^g \mathrm{e}^{-(\lambda _i + \mu _i)t} \prod _{k=0, k \ne i}^g \frac{1}{\lambda _k+\mu _k-\lambda _i-\mu _i}, \quad g\ge 1. \end{aligned} \end{aligned}$$

In this case the MS-G model becomes a birth-and-death process tracking cell generations, and becomes identical to that considered in Refs.^27,40–42^, where the inter-event times of cell death and division are modelled as exponential random variables, rather than Erlang distributions.

In this Section we consider the case with identical number of stages, *N*, and rates, $\lambda$ and $\mu$, for each generation, so that division times are Erlang-distributed in each generation. Under these assumptions, it is possible to obtain an analytical expression for the mean number of cells in each generation. Then Eq. (2.2) becomes

3.17 $$\begin{aligned} \frac{\mathrm{d}M^g_j(t)}{\mathrm{d}t}= {\left\{ \begin{array}{ll} -(\lambda + \mu ) M^0_1(t), &{} \text {if} \ g=0, \ j=1, \\ \lambda M^g_{j-1}(t) - (\lambda + \mu ) M^g_j(t), &{} \text {if} \ g \ge 0, \ j=2, \ldots , N, \\ 2 \lambda M^{g-1}_N(t) - (\lambda + \mu ) M^g_1(t), &{} \text {if} \ g \ge 1, \ j=1. \end{array}\right. } \end{aligned}$$

These equations can be rewritten in terms of the new variables $m^g_j(t) = \mathrm{e}^{(\lambda +\mu )t} M^g_j(t)$, for $g\ge 0$, $j=1,\ldots ,N$. This is equivalent to multiplying (3.17) by the integrating factor $e^{(\lambda +\mu )t}$. Thus, (3.17) becomes

3.18 $$\begin{aligned} \frac{\mathrm{d}m^g_j(t)}{\mathrm{d}t}= {\left\{ \begin{array}{ll} 0, &{} \text {if} \ g=0, \ j=1, \\ \lambda m^g_{j-1}(t), &{} \text {if} \ g \ge 0, \ j=2, \ldots , N, \\ 2 \lambda m^{g-1}_N(t), &{} \text {if} \ g \ge 1, \ j=1. \end{array}\right. } \end{aligned}$$

To determine the solutions of (3.18), we focus here on the case $M^0_1(0)=m^0_1(0)=C_0$ and all the other compartments are empty at time $t=0$. This implies that $m^0_1(t)=C_0$ for $t\ge 0$, and by solving (3.18) recursively one gets

$$\begin{aligned} m^0_j(t) = C_0 \lambda ^{j-1} \frac{t^{j-1}}{(j-1)!}, \ \ j=1, \ldots , N. \end{aligned}$$

This expression allows one then to determine the mean number of cells in each stage of generation 1,

3.19 $$\begin{aligned} m^1_j(t) = 2C_0\lambda ^{N + j-1} \frac{t^{N+j-1}}{(N+j-1)!}, \ \ j=1, \ldots , N. 
\end{aligned}$$

By recursion of (3.18) the mean number of cells in each compartment *j* of generation *g* is given by

$$\begin{aligned} m^g_j(t) = 2^g C_0 \lambda ^{gN+j-1} \frac{t^{gN+j-1}}{(gN+j-1)!} \ \text {for} \ g \ge 0, \ j=1, \dots , N. \end{aligned}$$

Going back to the original variables, $M^g_j(t)$, the solutions of (3.17) are

3.20 $$\begin{aligned} M^g_j(t) = 2^g C_0 \lambda ^{Ng+j-1} \frac{t^{Ng+j-1}}{(Ng+j-1)!} \mathrm{e}^{-(\lambda +\mu )t}, \ \ g \ge 0, \ j=1,\ldots ,N. \end{aligned}$$

From the previous equations, one can show that

$$\begin{aligned} \lim _{t \rightarrow +\infty } M^g_j(t) = 0, \; \text {for all} \ g \ge 0, \ j=1,\ldots ,N, \end{aligned}$$

since cells in each generation and compartment either proceed to the next stage within their generation, divide (proceeding to the next generation), or die.

Once the mean number of cells in each compartment for a given generation is at hand, the expected number of cells in each generation can be determined according to (2.3). We can write

3.21 $$\begin{aligned} M^g(t) = \sum _{j=1}^{N} M^g_j(t) = 2^g C_0 (\lambda t)^{Ng} \mathrm{e}^{-(\lambda +\mu )t} \sum _{j=1}^N \frac{(\lambda t)^{j-1}}{(Ng+j-1)!}, \ g \ge 0. \end{aligned}$$

This equation is consistent with the results of the exponential model ($N=1$)^27^. On the other hand, if one is interested in the mean number of cells in each compartment, $M_j(t)$ for $j=1,\dots ,N$, regardless of the generation they belong to, this can be computed as follows

3.22 $$\begin{aligned} \begin{aligned} M_j(t)&= \sum _{g=0}^{+\infty } M^g_j(t) = \sum _{g=0}^{+\infty } \; 2^g \ C_0 \ \lambda ^{gN+j-1} \; \frac{\mathrm{e}^{-( \lambda + \mu ) t} \ t^{gN+j-1}}{(gN+j-1)!}\\&= C_0 \; \mathrm{e}^{-( \lambda + \mu ) t} \; 2^{\frac{1-j}{N}} \; \sum _{g=0}^{+\infty } \; \frac{\left( 2^{\frac{1}{N}} \lambda t\right) ^{gN+j-1}}{(gN+j-1)!}, \end{aligned} \end{aligned}$$

for $j=1,\ldots ,N$ and $t\ge 0$. In practice, one could truncate the series above to get an approximation of the mean number of cells in each stage. However, we note that one can use instead the solution provided by (3.5), since the dynamics of the MS-G model is equivalent to the dynamics of the MS model, when the parameters *N*, $\lambda$ and $\mu$ are generation-independent. It can be numerically checked that this indeed provides equivalent results. In fact, when $N=1$ or $N=2$, one can analytically show the equivalence. In the former case ($N=1$), it is enough to recall the power series of the exponential function. In the latter case ($N=2$), we derive from (3.5)

$$\begin{aligned} M_1(t)&= \frac{C_0}{2} \mathrm{e}^{-(\lambda +\mu )t} \left( \mathrm{e}^{\sqrt{2} \lambda t} + \mathrm{e}^{- \sqrt{2} \lambda t} \right) ,\\ M_2(t)&= \frac{C_0}{2 \sqrt{2}} \mathrm{e}^{-(\lambda +\mu )t} \left( \mathrm{e}^{\sqrt{2} \lambda t} - \mathrm{e}^{- \sqrt{2} \lambda t} \right) , \end{aligned}$$

where we used the fact that $z=\mathrm{e}^{\pi i}=-1$. On the other hand, from (3.22) we obtain

$$\begin{aligned} M_1(t)&= C_0 \mathrm{e}^{-(\lambda +\mu )t} \cosh \left( \sqrt{2} \lambda t\right) = C_0 \mathrm{e}^{-(\lambda +\mu )t} \frac{\mathrm{e}^{\sqrt{2} \lambda t} + \mathrm{e}^{- \sqrt{2} \lambda t}}{2}, \\ M_2(t)&= C_0 \mathrm{e}^{-(\lambda +\mu )t} \sinh \left( \sqrt{2} \lambda t\right) = \frac{C_0}{\sqrt{2}} \mathrm{e}^{-(\lambda +\mu )t} \frac{\mathrm{e}^{\sqrt{2} \lambda t} - \mathrm{e}^{- \sqrt{2} \lambda t}}{2}. \end{aligned}$$

This shows that the two models lead to the same expected number of cells in each stage.

### Comparison between the MS-G model and the cyton model

The *cyton model* is a stochastic model proposed to describe the population dynamics of B and T lymphocytes^13^. Division and death times are regulated by two independent clocks, and the competition between both clocks determines the fate of the cell. When a cell divides, these clocks, which depend on the number of divisions the cell has undergone, are reset for each daughter cell. However, when analysing an in vitro experiment with this type of cells, there is evidence that not all cells either divide or die. For instance, a portion of them may not respond to the stimulation^43^, or may respond without division^44^. This is the reason why a progressor fraction is defined in the cyton model. This progressor fraction represents for a given generation, the fraction of cells that are capable of undergoing further division. Each clock is described by a probability density function, and the parameters that define these probabilities are the free parameters in the model. Right skewed distributions, such as log-normal or gamma, are usually adopted to characterise the two independent clocks that regulate cell division and death. In summary, the cyton model is based on the following assumptions:

- death and division are random events, characterised by a probability density function for the time to divide or die, respectively,
- these processes are independent, and compete to determine the fate of the cell,
- the clocks responsible for these processes are reset when a cell divides,
- only a fraction of the cells in each generation are capable to undergo further divisions, and
- the machineries that regulate cellular fate depend on the cell’s generation.

In order to translate these assumptions into mathematical terms, let $\gamma _g$ be the progressor fraction characterising cells having undergone *g* divisions, and let $\phi _g(\cdot )$ and $\psi _g(\cdot )$ represent the probability density functions for the time to division and death, respectively, for cells in generation *g*. The number of cells dividing for the first time, or dying, per unit time at time $t\ge 0$ can be calculated, respectively, as^13^:

3.23 $$\begin{aligned} n^{div}_{0}(t)&= \gamma _0 \; C_0 \left( 1- \int _0^t \psi _0(s) \mathrm{d}s\right) \phi _0(t), \end{aligned}$$

3.24 $$\begin{aligned} n^{die}_{0}(t)&= C_0 \left( 1- \gamma _0 \int _0^t \phi _0(s) \mathrm{d}s\right) \psi _0(t), \end{aligned}$$

where $C_0$ is the initial number of cells in the population. Consequently, the time evolution of the expected number of cells in generation 0, ${{{\widetilde{M}}}}^0(t)$, obeys the differential equation

3.25 $$\begin{aligned} \frac{\mathrm{d}{{{\widetilde{M}}}}^0(t)}{\mathrm{d}t} = -\left[ n^{div}_{0}(t) + n^{die}_{0}(t)\right] . \end{aligned}$$

The number of cells in generation *g* dividing, or dying, per unit time at time *t* can be computed, respectively, as

3.26 $$\begin{aligned} n^{div}_{g}(t)&= 2 \gamma _g \int _0^t n^{div}_{g-1}(s) \left[ 1- \int _0^{t-s} \psi _g(l) \mathrm{d}l\right] \phi _g(t-s) \mathrm{d}s, \end{aligned}$$

3.27 $$\begin{aligned} n^{die}_{g}(t)&= 2 \int _0^t n^{div}_{g-1}(s) \left[ 1- \gamma _g \int _0^{t-s} \phi _g(l) \mathrm{d}l\right] \psi _g(t-s) \mathrm{d}s. \end{aligned}$$

Hence, the dynamics of the average number of cells in each generation, ${{{\widetilde{M}}}}^g(t)$, is governed by the differential equations

3.28 $$\begin{aligned} \frac{\mathrm{d}{{{\widetilde{M}}}}^g(t)}{\mathrm{d}t} = 2 n^{div}_{g-1}(t) - n^{div}_{g}(t) - n^{die}_{g}(t), \ g \ge 1. \end{aligned}$$

In the next sections we show how the cyton model is equivalent to our model for particular choices of the probability density functions of the division and death clocks, $\phi _g(\cdot )$ and $\psi _g(\cdot )$, and the progressor faction $\gamma _g$.

#### Exponential time to division and death

We consider here the MS-G model with number of stages across generations equal to one, i.e., $N_g =1$ for all $g \ge 0$. This means that cells in generation *g* divide after an exponentially distributed time with rate $\lambda _g$, and die with rate $\mu _g$. We note that this is different to a standard Markov birth-and-death process, since rates are generation-dependent. Equation (2.4) become

3.29 $$\begin{aligned} \begin{aligned} \frac{\mathrm{d}M^0(t)}{\mathrm{d}t}&= -\left( \lambda _0+\mu _0\right) M^0(t), \\ \frac{\mathrm{d}M^g(t)}{\mathrm{d}t}&= 2 \lambda _{g-1} M^{g-1}(t) - \left( \lambda _g+\mu _g\right) M^g(t), \ g \ge 1. \end{aligned} \end{aligned}$$

In this case, our model is equivalent to the cyton model with exponential times for division and death, and progressor fraction $\gamma _g=1$, $g\ge 0$. One can show this equivalence by proving that $n^{div}_{g}(t) = \lambda _g M^g(t)$ and $n^{die}_{g}(t) = \mu _g M^g(t)$, by induction on *g*. In the cyton model, the assumption of exponential time to division and death implies that $\phi _g(t) = \lambda _g \mathrm{e}^{-\lambda _g t}$ and $\psi _g(t) = \mu _g \mathrm{e}^{-\mu _g t}$, $g \ge 0$. Therefore, according to (3.23) and (3.24), the number of cells at time *t* dividing for the first time or dying to exit generation 0 per unit time is given by

$$\begin{aligned} n^{div}_{0}(t) \ =\ C_0 \lambda _0 \mathrm{e}^{-(\lambda _0+\mu _0) t}, \quad n^{die}_{0}(t) \ = \ C_0 \mu _0 \mathrm{e}^{-(\lambda _0+\mu _0) t}. \end{aligned}$$

We know from (3.16) that $M^0(t) = C_0 \mathrm{e}^{-(\lambda _0+\mu _0) t}$. Therefore, we can write $n^{div}_{0}(t)= \lambda _0 M^0(t)$ and $n^{die}_{0}(t)= \mu _0 M^0(t)$, which proves the case $g=0$. We assume $n^{div}_{g}(t) = \lambda _g M^g(t)$ and $n^{die}_{g}(t) = \mu _g M^g(t)$ hold for generation *g* and we need to show they also hold for generation $g+1$. We make use of (3.16) and (3.26) to write

$$\begin{aligned} n^{div}_{g+1}(t)&= 2 \int _0^t \lambda _g 2^g C_0 \prod _{l=0}^{g-1} \lambda _l \sum _{i=0}^g \mathrm{e}^{-(\lambda _i + \mu _i)s} \prod _{k=0, k \ne i}^g \frac{1}{\lambda _k+\mu _k-\lambda _i-\mu _i} \lambda _{g+1} \mathrm{e}^{-(\lambda _{g+1} + \mu _{g+1})(t-s)} \mathrm{d}s\\&= \lambda _{g+1} 2^{g+1} C_0 \prod _{l=0}^g \lambda _l \sum _{i=0}^g \mathrm{e}^{-(\lambda _{g+1} + \mu _{g+1})t} \int _0^t \prod _{k=0, k \ne i}^g \frac{\mathrm{e}^{(\lambda _{g+1} + \mu _{g+1} - \lambda _g -\mu _g)s}}{\lambda _k+\mu _k-\lambda _i-\mu _i} \mathrm{d}s\\&= \lambda _{g+1} M^{g+1}(t). \end{aligned}$$

For the number of cells in generation $g+1$ dying, Eq. (3.27), together with (3.16) lead to

$$\begin{aligned} n^{die}_{g+1}(t)&= 2 \int _0^t \lambda _g 2^g C_0 \prod _{l=0}^{g-1} \lambda _l \sum _{i=0}^g \mathrm{e}^{-(\lambda _i + \mu _i)s} \prod _{k=0, k \ne i}^g \frac{1}{\lambda _k+\mu _k-\lambda _i-\mu _i} \mu _{g+1} \mathrm{e}^{-(\lambda _{g+1} + \mu _{g+1})(t-s)} \mathrm{d}s\\&= \mu _{g+1} 2^{g+1} C_0 \prod _{l=0}^g \lambda _l \sum _{i=0}^g \mathrm{e}^{-(\lambda _{g+1} + \mu _{g+1})t} \int _0^t \prod _{k=0, k \ne i}^g \frac{\mathrm{e}^{(\lambda _{g+1} + \mu _{g+1} - \lambda _g -\mu _g)s}}{\lambda _k+\mu _k-\lambda _i-\mu _i} \mathrm{d}s\\&= \mu _{g+1} M^{g+1}(t), \end{aligned}$$

which concludes the proof. With the identities $n^{div}_{g}(t) = \lambda _g M^g(t)$ and $n^{die}_{g}(t) = \mu _g M^g(t)$ in (3.25) and (3.28), one can show that $M^g(t)$ and ${{{\widetilde{M}}}}^g(t)$ obey the same differential equations for all $g \ge 0$. Thus, the two models are equivalent.

#### Erlang time to division and exponential time to death

We now consider the more interesting case where the number of stages in each generation is greater than one, and the cell cycle can be described as a multi-stage process. We focus here on the case where identical number of stages *N* and birth and death rates, $\lambda$ and $\mu$, respectively, are considered across generations. Similarly to the previous case, we prove that $n^{div}_{g}(t) = \lambda M^g_N(t)$ and $n^{die}_{g}(t) = \mu M^g(t)$ by induction on *g*. Since a cell’s time to division is Erlang distributed and a cell’s time to death is exponentially distributed, $\psi _g(t) = \mu \mathrm{e}^{-\mu t}$ for all $g \ge 0$ and

$$\begin{aligned} \phi _g(t) = \frac{\lambda ^N t^{N-1} \mathrm{e}^{-\lambda t}}{(N-1)!}, \ g \ge 0, \end{aligned}$$

where the progressor fraction is again set to 1 for each generation. Note that in this case the parameters in $\phi _g(\cdot )$ and $\psi _g(\cdot )$ are independent of the generation *g*, since the number of stages and the birth and death rates are identical for all generations. From (3.23) and (3.24), the number of cells dividing for the first time or dying to exit generation 0 per unit time at time *t* is

$$\begin{aligned} n^{div}_{0}(t) \ =\ \frac{C_0 \lambda ^N t^{N-1}}{(N-1)!} \mathrm{e}^{-(\lambda +\mu ) t}, \quad n^{die}_{0}(t) \ =\ C_0 \mu \mathrm{e}^{-(\lambda +\mu ) t} \sum _{j=0}^{N-1} \frac{(\lambda t)^j}{j!}. \end{aligned}$$

The dynamics of the expected number of cells in generation 0 is given by (3.25), as in the previous case. Using (3.20) and (3.21), we observe that

$$\begin{aligned} M^0(t) \ =\ C_0 \mathrm{e}^{-(\lambda +\mu ) t} \sum _{j=0}^{N-1} \frac{(\lambda t)^j}{j!}, \quad M^0_N(t) \ =\ \frac{\lambda ^N t^{N-1}}{(N-1)!} \mathrm{e}^{-(\lambda +\mu ) t}. \end{aligned}$$

Therefore, $n^{div}_{0}(t)= \lambda M^0_N(t)$ and $n^{die}_{0}(t)= \mu M^0(t)$, which concludes the case $g=0$. We make use of these identities in (3.25) to obtain

$$\begin{aligned} \frac{\mathrm{d}{{{\widetilde{M}}}}^0(t)}{\mathrm{d}t} = -\lambda M^0_N(t) - \mu M^0(t), \end{aligned}$$

which is the differential equation derived in (2.4) for $M^0(t)$. Now, let us suppose that the identities $n^{div}_{g}(t) = \lambda M^g_N(t)$ and $n^{die}_{g}(t) = \mu M^g(t)$ hold for generation *g* and we prove them for generation $g+1$. Using (3.26) and the induction hypothesis, we have

$$\begin{aligned} n^{div}_{g+1}(t)&= 2 \int _0^t \lambda 2^g C_0 \frac{(\lambda s)^{Ng + N -1}}{(Ng + N -1)!} \mathrm{e}^{-(\lambda + \mu )s} \mathrm{e}^{-\mu (t-s)} \frac{\lambda ^N (t-s)^{N-1} \mathrm{e}^{-\lambda (t-s)}}{(N -1)!} \mathrm{d}s\\&= 2^{g+1} \frac{\lambda ^{N(g+2)}}{(N(g+1)-1)!} C_0 \mathrm{e}^{-(\lambda + \mu )t} \frac{1}{(N-1)!} \int _0^t s^{N(g+1)-1} (t-s)^{N-1} \mathrm{d}s\\&= 2^{g+1} \frac{\lambda ^{N(g+2)}}{(N(g+1)-1)!} C_0 \mathrm{e}^{-(\lambda + \mu )t} \sum _{j=0}^{N-1} \frac{(-1)^j t^{N-1-j}}{j! (N-1-j)!} \int _0^t s^{N(g+1)-1+j}ds \\&= \lambda 2^{g+1} \frac{(\lambda t)^{N(g+1)+N-1}}{(N(g+1)+N-1)!} C_0 \mathrm{e}^{-(\lambda + \mu )t} \ =\ \lambda M^{g+1}_N(t), \end{aligned}$$

where we have used (3.20) for the last step. The same arguments can be used to look at the number of cells in generation $g+1$ dying per unit of time,  (3.27). Together with the induction hypothesis, we can write

$$\begin{aligned} n^{die}_{g+1}(t)&= 2 \int _0^t \lambda 2^g C_0 \frac{(\lambda s)^{Ng + N -1}}{(Ng + N -1)!} \mathrm{e}^{-(\lambda + \mu )s} \mathrm{e}^{-\lambda (t-s)} \sum _{j=0}^{N-1} \frac{\lambda ^j (t-s)^j}{j!} \mu \mathrm{e}^{-\mu (t-s)} \mathrm{d}s\\&= 2^{g+1} \lambda ^{Ng + N} C_0 \frac{\mathrm{e}^{-(\lambda + \mu )t} \mu }{(Ng + N -1)!} \sum _{j=0}^{N-1} \frac{\lambda ^j}{j!} \int _0^t s^{Ng + N - 1} (t-s)^j \mathrm{d}s\\&= 2^{g+1} \lambda ^{Ng + N} C_0 \mathrm{e}^{-(\lambda + \mu )t} \mu \sum _{j=0}^{N-1} \lambda ^j \sum _{k=0}^j \frac{t^j}{k!(j-k)!} \frac{t^{k+N+Ng}}{k+N+Ng} \frac{(-1)^k}{(Ng+N-1)!} \\&= \mu 2^{g+1} C_0 \mathrm{e}^{-(\lambda + \mu )t} \sum _{j=0}^{N-1} \frac{(\lambda t)^{N(g+1)+j}}{(N(g+1)+j)!} \ = \ \mu M^{g+1}(t), 
\end{aligned}$$

where the last identity was obtained making use of (3.21). Hence, (3.28) becomes

$$\begin{aligned} \frac{\mathrm{d}{{{\widetilde{M}}}}^g(t)}{\mathrm{d}t} = 2 \lambda M^{g-1}_N(t) - \lambda M^g_N(t) - \mu M^g(t), \ g \ge 1, \end{aligned}$$

which is identical to (2.4) for $M^g(t)$, $g \ge 1$. This concludes the proof of the equivalence between the cyton model and the multi-stage model with generations when a cell’s time to divide is Erlang distributed with parameters $\lambda$ and *N*, and a cell’s time to die is exponential with rate $\mu$. In summary, the analysis presented in this section for the multi-stage model with Erlang division time and exponential death time leads to exact closed solutions for the cyton model with the previous choice of clocks.

## Case study: lymphopenia-induced proliferation

In this Section we illustrate the applicability of the MS-G model to CFSE data, making use of an experimental study of lymphopenia-induced proliferation^20^. In particular, we compare the performance of the MS-G model to that of a simple exponential (or single stage) model with generations, which is equivalent to making $N_g=1$ for all *g* in the MS-G model.

**Figure 5:**
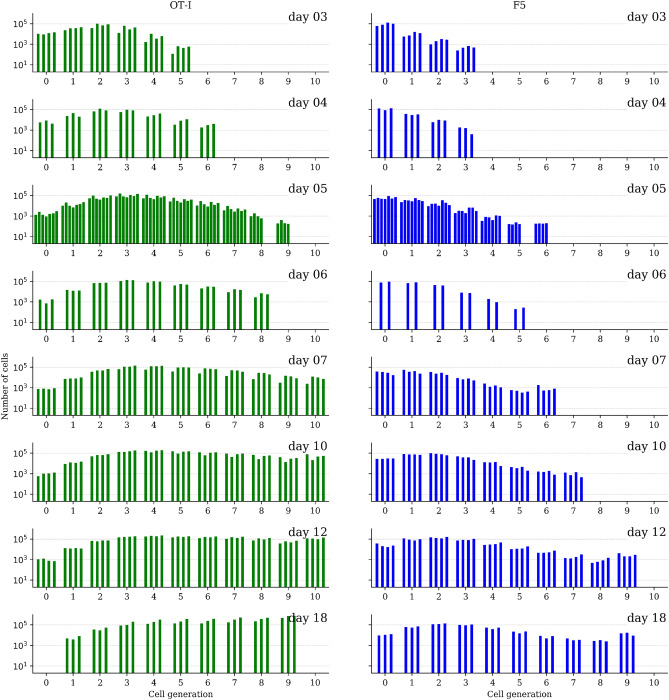
Data set of murine T lymphocytes from Hogan et al.^20^. Left: OT-I T cells. Right: F5 T cells. For each time point, the number of cells is plotted for each mouse and generation.

Differences in T cell proliferation have been observed to vary between different T cell clonotypes (i.e., the set of T cells with the same T cell receptor). Hogan et al.^20^ transferred CFSE-labelled OT-I or F5 T cells intravenously to lymphopenic mice. A certain number of days (3, 4, 5, 6, 7, 10, 12 and 18 days) after the transfer, spleens and lymph nodes were recovered from the mice and analysed by flow cytometry to quantify the expression levels of CD8, CD5, CD44, and CFSE dilution^20^. For each time point, the number of mice analysed was between 3 and 7. We note that two independent transfer experiments, carried out under identical conditions, were performed: one for OT-I cells and a second one for F5. In Fig. 5 both data sets are shown: for each time point the number of cells is plotted for each mouse and generation (identified via the CFSE dilution measurement). On the left (right), OT-I (F5) cells are represented by the green (blue) histograms. In order to infer model parameters, we will consider all cells which have divided five or more times as a single class, denoted $5+$. This is similar to the approach considered in Refs.^4,29,35^. The rationale behind this choice is to reduce errors in the quantification of labelled cells with low CFSE fluorescence, as is the case for five or more divisions.

Figure 5 clearly shows that OT-I T cells proliferate faster than F5 cells, so that by day 7 there are OT-I cells in generation 10, whereas for F5 cells the maximum generation observed at day 7 is 6. This greater proliferative capacity of OT-I cells eventually leads, after one week, to competition for resources (e.g., IL-7 cytokine) and the OT-I population approaching its carrying capacity^20^. Since our model does not account for competition, it can only appropriately describe the dynamics of OT-I cells during the first week of the experiment. Thus, for OT-I cells we will only make use of the data set up to that time (one week). Yet for the F5 population we will use the entire data set. In Hogan et al.^20^ this competition was incorporated with a density-dependent birth rate, $\lambda (P)$, as follows

**Figure 6:**
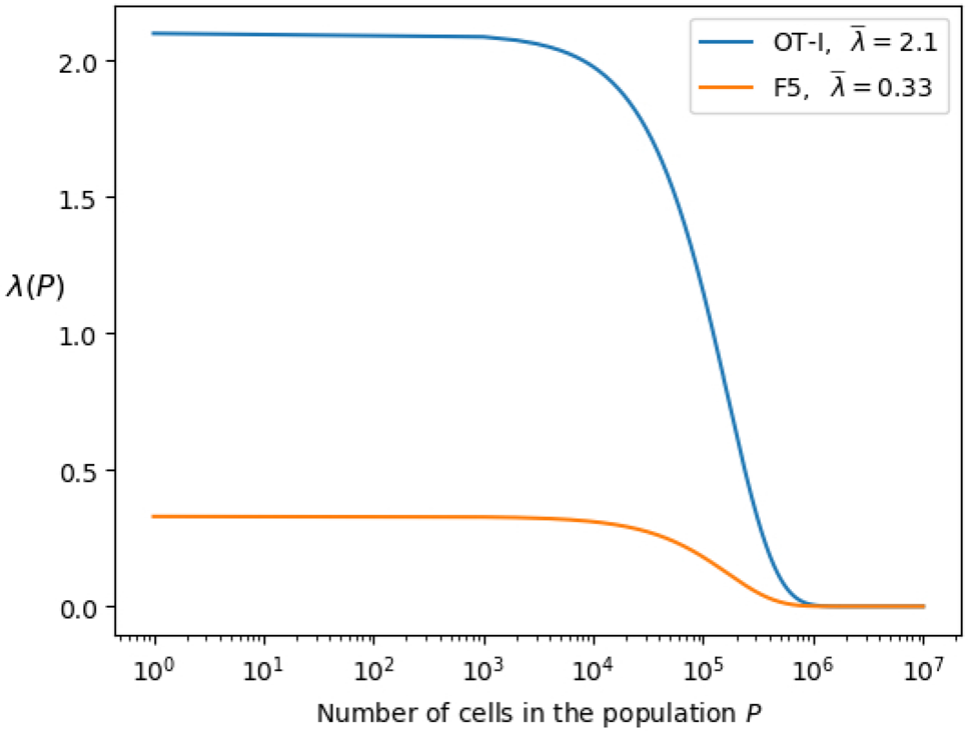
Density-dependent birth rate, $\lambda (P)$, as a function of the population size, *P*. The parameter ${{\bar{\lambda }}}$, with units of $cell \cdot day^{-1}$, represents the rate of growth under no competition and $\delta$ quantifies the level of reduction caused by the expansion of competing cells. Values for ${{\bar{\lambda }}}$ (shown in the inset) and $\delta =6.0 \times 10^{-6}$ are taken from^20^, Table 1].

4.1 $$\begin{aligned} \lambda (P) = {{\bar{\lambda }}} \; \mathrm{e}^{-\delta P}, \end{aligned}$$

where ${{\bar{\lambda }}}$ is the rate of growth under unlimited resources, $\delta$ the size of reduction caused by the expansion of competing cells, and *P* is the size of the population^20^. Figure 6 shows the density-dependent birth rate, $\lambda (P)$, as a function of the population size *P*. It suggests that the competition for resources is greater in the case of OT-I T cells. In the experiments the number of OT-I cells after one week (about $5 \times 10^5$) is larger than the population of F5 T cells at day 18 (about $4 \times 10^5$). Therefore, the population of F5 T cells never reaches its carrying capacity and the role of competition for resources can be neglected.

We estimate model parameters with the ABC-SMC algorithm^33^. Thus, the posterior distribution of the parameters is obtained by *T* sequential applications of the ABC algorithm, where the posterior obtained in each iteration is used as prior for the next one. This algorithm requires the definition of prior distributions for the first iteration, a distance function, a tolerance threshold for each iteration, and a perturbation kernel^33^. We assume all parameters are initially distributed according to a uniform prior distribution, as described in Table 1. When a prior distribution spans several orders of magnitude, the uniform distribution is taken over the exponent to efficiently explore parameter space. Given $x_D^g(t)$, the experimentally determined mean number of cells in generation *g* at time *t*, for $g\in \{0,1,2,3,4,5+\}$, and its corresponding model prediction, $x^{g}_M(t)=M^g(t)$ for a particular choice of parameters ${\varvec{\theta }}=(C_0,N_0,N,\lambda _0,\lambda ,\alpha )$, the distance function is defined as

4.2 $$\begin{aligned} d(\text {model,data} \ | \ {\varvec{\theta }}) = \sqrt{\sum _{g=0}^{G} \sum _{t \in {\mathscr {T}}} \left[  \frac{x^{g}_M(t) - x_D^g(t)}{\sigma _D^g(t)} \right] ^2}, \end{aligned}$$

where ${\mathscr {T}}$ is the set of time points and depends on the clonotype of interest, $\sigma _D^g(t)$ represents the standard deviation of the experimental data at time *t* and generation *g*, and *G* is the merged (and maximum) generation, $G=5+$. In practice, we define the first tolerance threshold, $\varepsilon _1$, in the ABC-SMC algorithm as the median value of the distances obtained from $10^4$ preliminary realisations, with the parameters sampled from the prior distributions in Table 1. The subsequent tolerance thresholds, $\varepsilon _j$, $j=2,\ldots ,T$ can be then defined as the median of the distance values obtained from the previous iterations of the algorithm. Finally, we use a uniform perturbation kernel to perturb the parameters during the sequence of iterations^33^, and implement the algorithm for $T= 16$ in the case of the multi-stage model and $T=7$ for the single stage one.

Before performing the Bayesian inference, we make some assumptions based on the experimental set up. Several studies have shown that the time to a first division is larger the time to subsequent divisions, since cells require time to become activated before they divide^13,15,16^. Thus, we assume that all generations but 0 are comprised of the same number of stages *N*, whereas generation 0 is characterised by $N_0$ stages. Similarly, cells in generation 0 proceed to divide with birth rate $\lambda _0$, whilst all the other generations have a birth rate $\lambda$. Therefore, in contrast to the inference in^29^, the number of stages $N_0$ and *N* are free parameters in the model. On the other hand, we propose that the per cell death rate in a given generation is linear on the number of cell divisions that the cell has undergone^35,45^. We write

4.3 $$\begin{aligned} \mu _g = \alpha \; g, \ g \ge 0, \end{aligned}$$

where $\alpha$ is a parameter to estimate. These linear death rates encode the fact that cells are more likely to die when they have already undergone several divisions^35,45^. Finally, the initial number of cells, $C_0$, is considered a parameter to be estimated, since the actual number of transferred cells which make it to the lymph nodes or spleen cannot be measured.

**Table 1:** Prior distributions for model parameters. Units for $\lambda _0$, $\lambda$ and $\alpha$ are inverse hours ($h^{-1}$).

Model parameters	Description	Prior distribution
$C_0$	Initial number of cells	$C_0=10^x$, $x\sim U(4,6)$
$N_0$, *N*	Number of stages	$U_{\text {discrete}}(1,50)$
$\lambda _0$, $\lambda$	Birth rate	$\lambda _0=10^y$, $\lambda =10^z$, $y,z\sim U(-3,1)$
$\alpha$	Death rate slope	$\alpha =10^w$, $w\sim U(-5,-1)$

**Figure 7:**
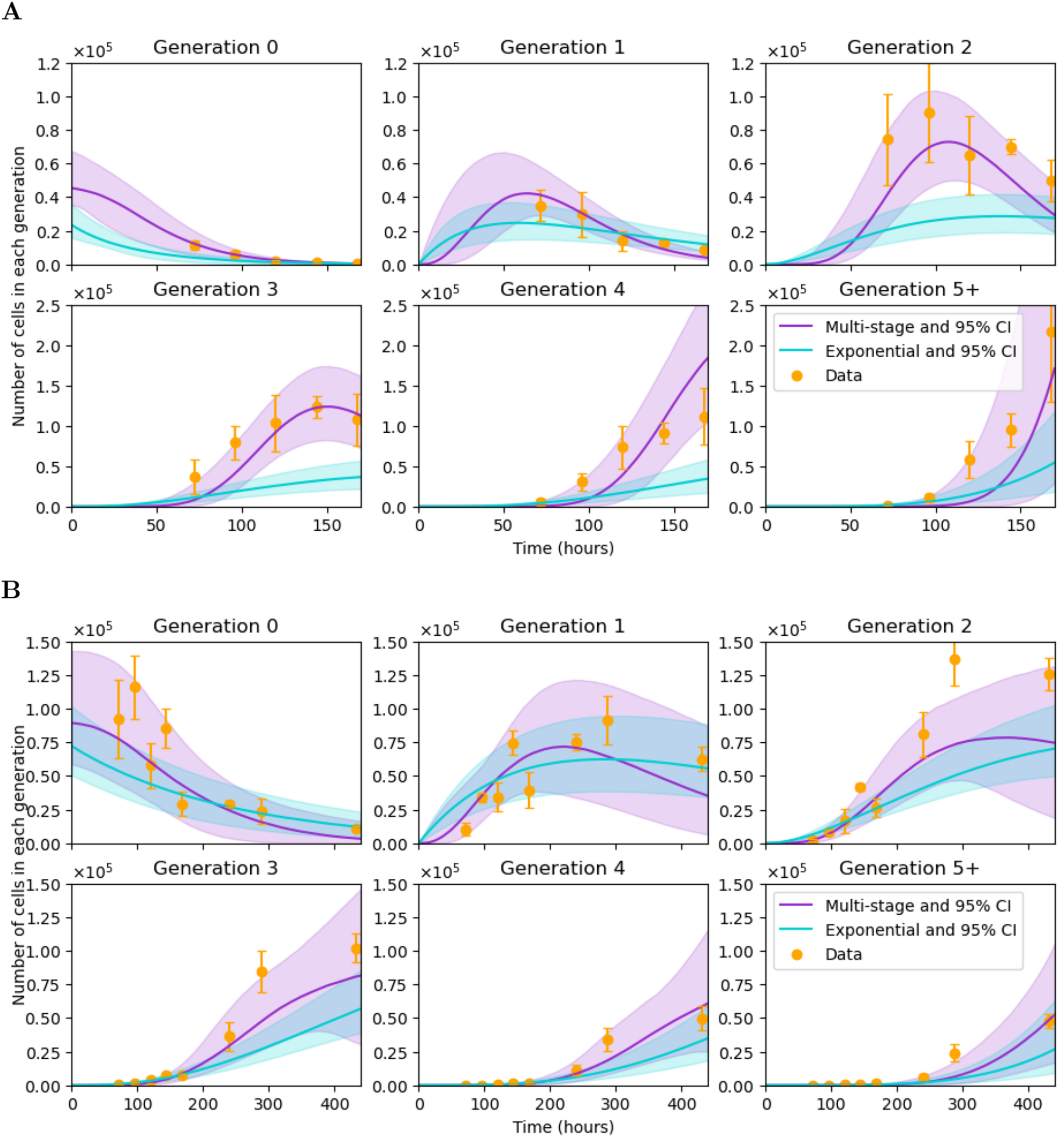
Exponential (solid turquoise line) and multi-stage (solid magenta line) model predictions compared to the data sets (orange dots) for OT-I (**A**) and F5 (**B**) T cells. Bars on data points represent their standard deviation. The expected number of cells in each generation is plotted as a function of time. These predictions represent the median value of $10^4$ simulations with the accepted parameter values from the posterior distributions. Shaded areas represent 95$\%$ confidence intervals.

The predictions obtained for each model, and for each clonotype (OT-I or F5), are shown in Fig. 7. We run the model with the parameters being sampled from the estimated posterior distributions and compute the median of all the simulations, which corresponds to the solid magenta (multi-stage model) and turquoise (exponential model) lines in Fig. 7. The bands around median predictions represent 95$\%$ confidence intervals. Data points are plotted with the standard deviation from the multiple experimental replicates. As shown in Fig. 7, the calibrated MS-G model successfully captures the dynamics of the proliferating T cell populations (OT-I and F5), whereas the single stage model significantly underestimates the expected number of cells beyond generation 1, particularly in the case of OT-I T cells. We made use of the corrected version of the Akaike Information Criterion ($\hbox {AIC}_C$)^46,47^ to quantify the better fit of the MS-G model accounting for the extra parameters $N_0$ and *N* in the multi-stage model. The values of $\hbox {AIC}_C$ for each model and clonotype are listed in Table 2. Despite the two extra parameters, the values of $\hbox {AIC}_C$ corresponding to the multi-stage model are significantly lower for both clonotypes. Overall, the MS-G model is able to explain the data from the OT-I transfer experiment better, since this data set is less noisy than the F5 set.

**Table 2:** $\hbox {AIC}_C$ values for the exponential and multi-stage models calibrated with CFSE data of murine T lymphocytes.

Mathematical model	Cell type	Value of $\hbox {AIC}_C$
Multi-stage	OT-I T cells	50.4
Exponential	OT-I T cells	283
Multi-stage	F5 T cells	206
Exponential	F5 T cells	317

**Figure 8:**
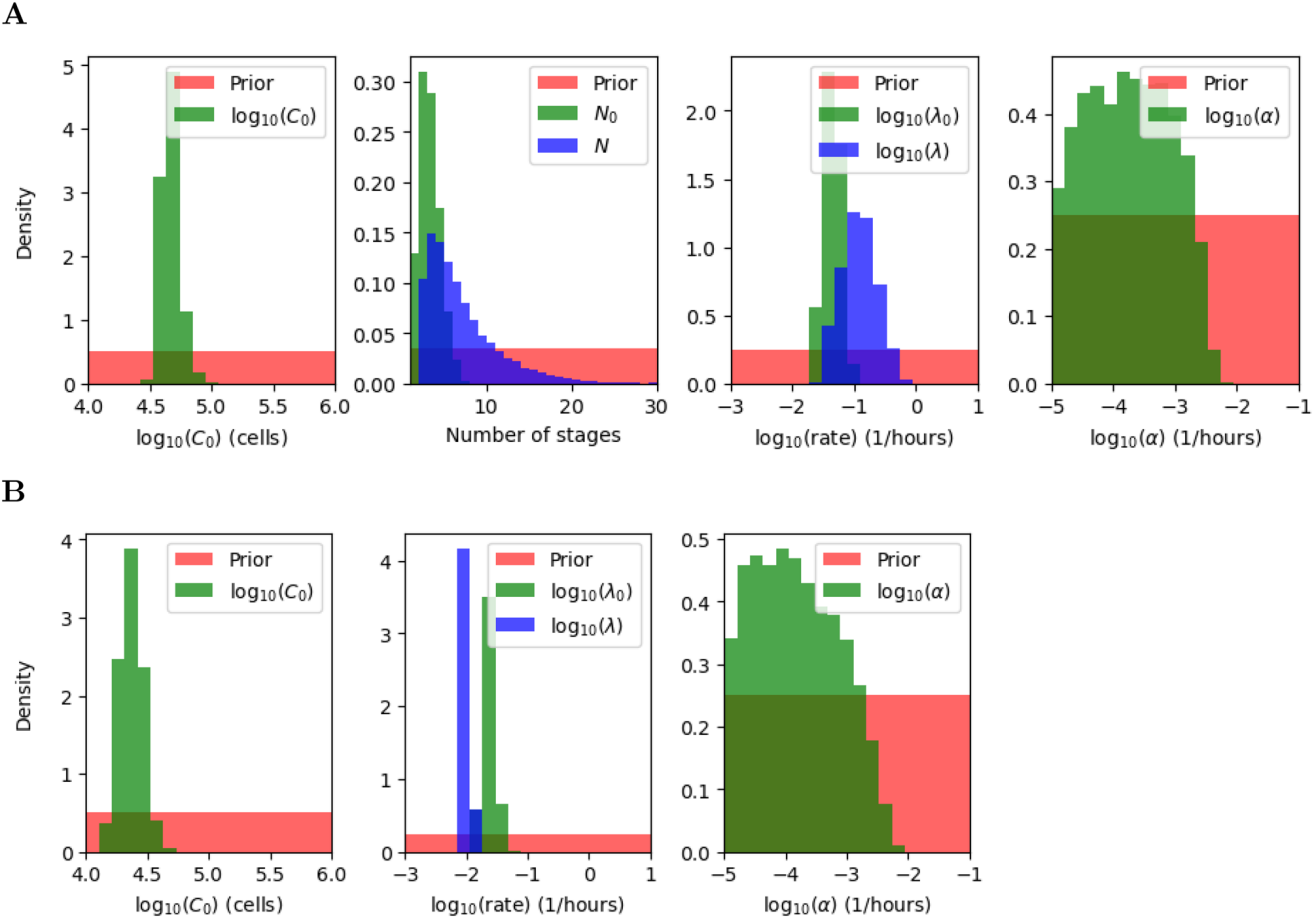
Posterior distributions (green and blue) for the parameters in the multi-stage (**A**) and exponential (**B**) model for OT-I T cells. In the exponential model, the number of stages for all generations is equal to 1, i.e., $N_0 = N = 1$. Prior distributions are shown in red.

**Figure 9:**
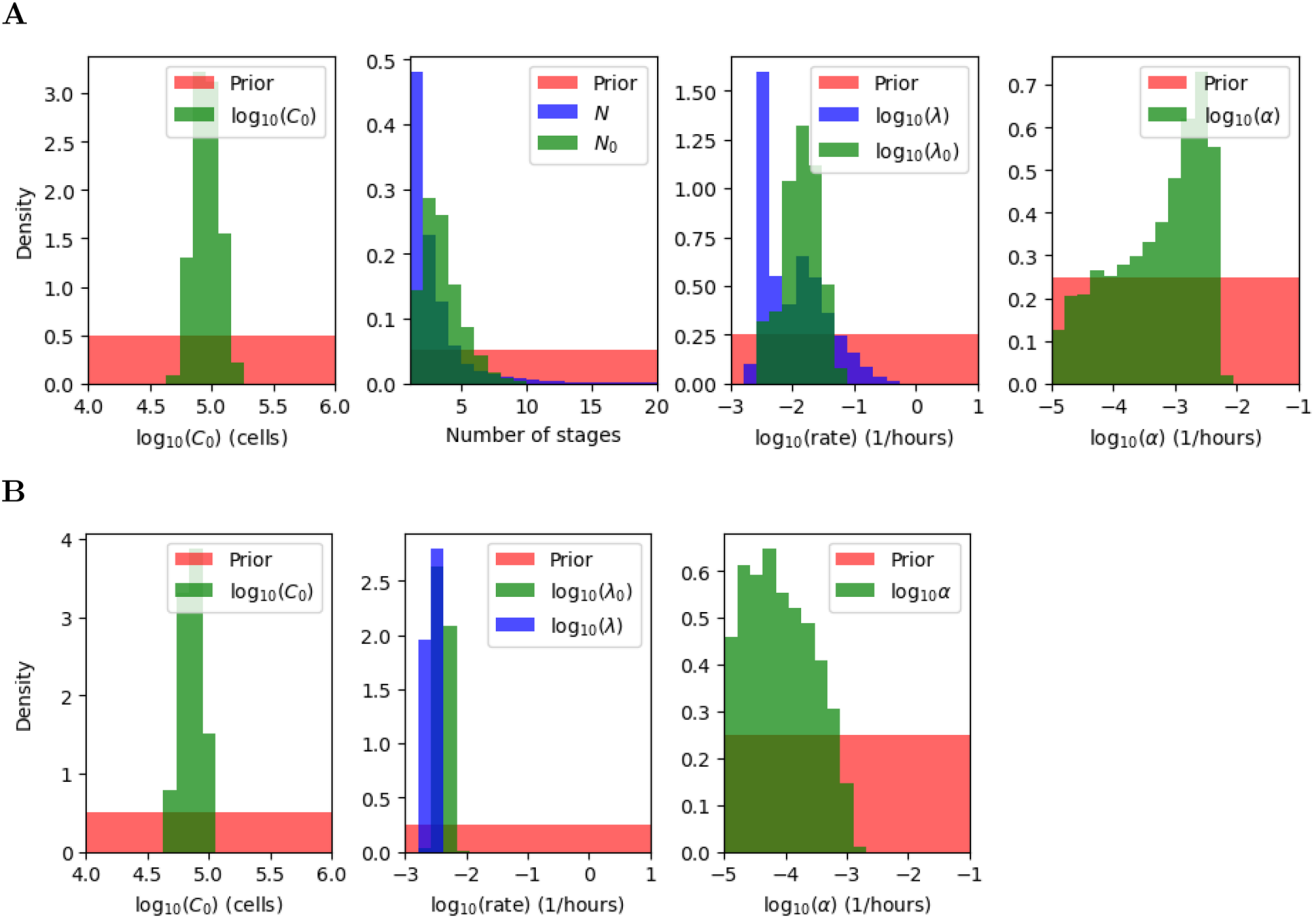
Posterior distributions (green and blue) for the parameters in the multi-stage (**A**) and exponential (**B**) model for F5 T cells. In the exponential model, the number of stages for all generations is equal to 1, i.e., $N_0 = N = 1$. Prior distributions are shown in red.

**Figure 10:**
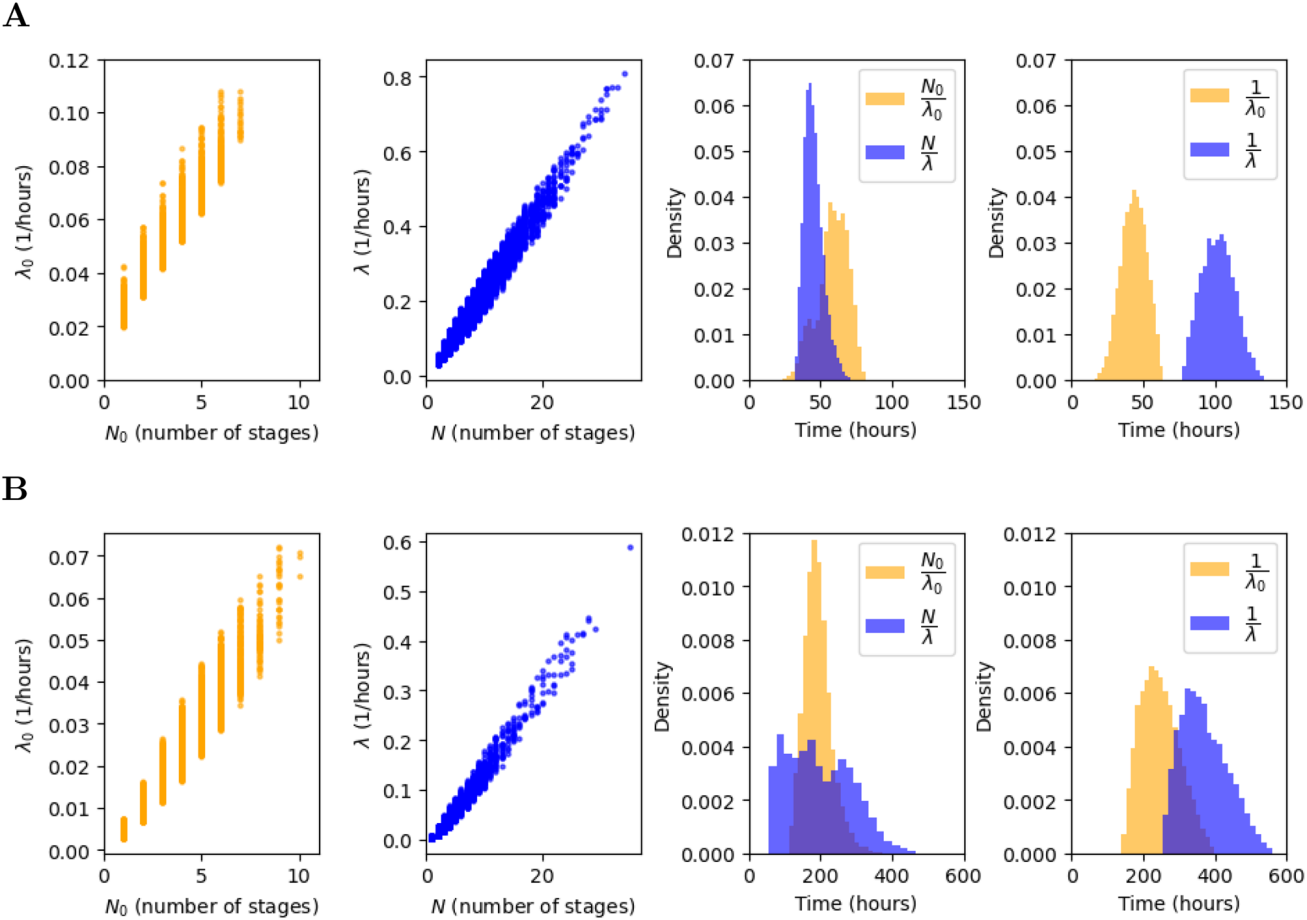
Joint posterior distributions (left two plots) of the number of stages $N_0$, *N* and the birth rates $\lambda _0$, $\lambda$. Marginal posterior distributions (right two plots) for the mean time to first and subsequent divisions estimated from the multi-stage model (third column) and the exponential model (fourth column). Panel **A** for OT-I T cells and **B** for F5 T cells.

The marginal posterior distributions for each parameter are shown in green and blue in Figs. 8 and 9, for the multi-stage and exponential models, respectively, and the (uniform) prior distributions are plotted in red. Summary statistics of these posterior distributions are shown in Tables 3, 4, 5 and 6. Cell death is governed by the parameter $\alpha$, and is estimated to be low for both models and clonotypes, suggesting that cell death does not have a significant impact on the dynamics during lymphopenia, which is in fact dominated by cell division. This result is in agreement with Hogan et al.^20^, where the death rate is assumed to be zero. The initial number of cells can be estimated with relative success, and does not seem to depend heavily on the model considered. On the other hand, cell division is governed by parameters $(N_0,\lambda _0,N,\lambda )$, with $N_0=N=1$ in the exponential model. We note that in both models, $\frac{N_0}{\lambda _0}$ and $\frac{N}{\lambda }$ represent the mean time to the first and subsequent divisions, respectively. Although all division-related parameters can be estimated from the data, for both models and clonotypes, a correlation between the division rate and the number of stages is seen in the scatter plots of Fig. 10. Instead of plotting the marginal posterior distributions for these parameters, one can consider the posterior distribution for the mean times $\frac{N_0}{\lambda _0}$ and $\frac{N}{\lambda }$ (see Fig. 10). The fact that $N=1$ is never chosen as an accepted parameter value in the posterior distribution for the multi-stage model and the OT-I clonotype already suggests that a multi-stage representation of cell division is preferred for this clonotype. On the other hand for the F5 clonotype the marginal distribution for *N* shows a non-zero frequency for the value 1, but larger values of *N* are also represented in its posterior distribution. The mean time to both first and subsequent divisions, $\frac{N_0}{\lambda _0}$ and $\frac{N}{\lambda }$, are significantly longer for the F5 clonotype than the OT-I. In fact, our results estimate that F5 T cells divide slowly compared to OT-I cells, requiring on average 192 h to carry out a first division (59 h taken by OT-I T cells), as shown in Fig. 10 for the multi-stage model. The time to subsequent divisions is represented by the blue histograms. Interestingly, our estimation of the mean time to first division of OT-I cells, on average 59 h, is close to the value obtained by Hogan et al.^20^ (52 h when considering the best fit parameter estimates). In the case of F5 cells, we predict an average of 192 h to undergo their first division, whereas Hogan et al. obtained a value of 137 h. We note that the value 137 h is within the range covered by our predicted posterior distribution.

**Table 3:** Summary statistics of OT-I clonotype posterior distributions for the multi-stage model.

Parameter	Minimum	Maximum	Mean	Median	Standard deviation
$C_0$	$3.00 \times 10^4$	$1.03 \times 10^5$	$4.67 \times 10^4$	$4.54 \times 10^4$	$8.03 \times 10^3$
$N_0$	1	7	2.83	3	1.23
*N*	2	34	6.59	5	4.30
$\lambda _0$	$1.98 \times 10^{-2}$	$1.08 \times 10^{-1}$	$4.64 \times 10^{-2}$	$4.56 \times 10^{-2}$	$1.45 \times 10^{-2}$
$\lambda$	$2.80 \times 10^{-2}$	$8.08 \times 10^{-1}$	$1.48 \times 10^{-1}$	$1.20 \times 10^{-1}$	$1.01 \times 10^{-1}$
$\alpha$	$1.00 \times 10^{-5}$	$5.97 \times 10^{-3}$	$5.06 \times 10^{-4}$	$1.76 \times 10^{-4}$	$7.47 \times 10^{-4}$

**Table 4:** Summary statistics of F5 clonotype posterior distributions for the multi-stage model.

Parameter	Minimum	Maximum	Mean	Median	Standard deviation
$C_0$	$4.74 \times 10^4$	$1.85 \times 10^5$	$9.26 \times 10^4$	$8.94 \times 10^4$	$2.22 \times 10^4$
$N_0$	1	10	3.01	3	1.53
*N*	1	35	2.42	2	2.57
$\lambda _0$	$2.68 \times 10^{-3}$	$7.20 \times 10^{-2}$	$1.70 \times 10^{-2}$	$1.47 \times 10^{-2}$	$1.07 \times 10^{-2}$
$\lambda$	$2.06 \times 10^{-3}$	$5.88 \times 10^{-1}$	$2.20 \times 10^{-2}$	$9.54 \times 10^{-3}$	$3.90 \times 10^{-2}$
$\alpha$	$1.00 \times 10^{-5}$	$6.21 \times 10^{-3}$	$1.35 \times 10^{-3}$	$8.19 \times 10^{-4}$	$1.40 \times 10^{-3}$

**Table 5:** Summary statistics for the posterior distributions of the exponential model for the OT-I clonotype.

Parameter	Minimum	Maximum	Mean	Median	Standard deviation
$C_0$	$1.43 \times 10^4$	$5.46 \times 10^5$	$2.40 \times 10^4$	$2.34 \times 10^4$	$5.28 \times 10^3$
$\lambda _0$	$1.56 \times 10^{-2}$	$6.19 \times 10^{-2}$	$2.40 \times 10^{-2}$	$2.28 \times 10^{-2}$	$5.69 \times 10^{-3}$
$\lambda$	$7.45 \times 10^{-3}$	$1.29 \times 10^{-2}$	$9.88 \times 10^{-3}$	$9.78 \times 10^{-3}$	$1.10 \times 10^{-3}$
$\alpha$	$1.00 \times 10^{-5}$	$7.23 \times 10^{-3}$	$4.76 \times 10^{-4}$	$1.32 \times 10^{-4}$	$8.17 \times 10^{-4}$

**Table 6:** Summary statistics for the posterior distributions of the exponential model for the F5 clonotype.

Parameter	Minimum	Maximum	Mean	Median	Standard deviation
$C_0$	$4.54 \times 10^3$	$1.10 \times 10^5$	$7.33 \times 10^4$	$7.22 \times 10^4$	$1.39 \times 10^4$
$\lambda _0$	$2.50 \times 10^{-3}$	$7.18 \times 10^{-3}$	$4.24 \times 10^{-3}$	$4.13 \times 10^{-3}$	$9.28 \times 10^{-4}$
$\lambda$	$1.79 \times 10^{-3}$	$3.92 \times 10^{-3}$	$2.77 \times 10^{-3}$	$2.76 \times 10^{-3}$	$4.66 \times 10^{-4}$
$\alpha$	$1.00 \times 10^{-5}$	$1.52 \times 10^{-3}$	$1.72 \times 10^{-4}$	$7.30 \times 10^{-5}$	$2.23 \times 10^{-4}$

Our results indicate that OT-I T lymphocytes require on average 59 h for their first division, and a bit less, 46 h, for subsequent divisions (see upper left plot of Fig. 10). Based on our Bayesian approach, we conclude that a multi-stage model with a constant division rate after the first division event, is a suitable description of lymphopenia-induced proliferation^5,29,40^. The MS-G model estimates that F5 cells take on average slightly less than 200 h to divide, both for the first or subsequent division rounds, as shown in the lower left plot of Fig. 10. This difference can be explained by the different characteristics of OT-I and F5 T cells, and was previously observed^20^. The posterior distributions of the expected time to subsequent divisions in the MS-G model, $\frac{N}{\lambda }$, and in the exponential one $\frac{1}{\lambda }$, shown as blue histograms in Fig. 10, indicate that the exponential model predicts a longer division time than the multi-stage model for both clonotypes. This can be justified by the implementation of the ABC-SMC algorithm. Indeed, when parameterising the exponential model, the algorithm tries to keep the distance between the model predictions and the experimental observations low. This leads to the choice of parameter sets which limit cell proliferation, as shorter division times in the exponential model would lead to an increase in cell numbers not observed in the data set, and thus larger distance values. This is why the estimated birth rates in the exponential model are lower than the ones in the multi-stage representation. As a result, the exponential model predicts a greater average division time than the multi-stage model for both clonotypes. Finally, our results indicate that for both clonotypes the exponential model (see Fig. 10) found a shorter time to first division than to subsequent ones, contradicting previous findings^13,15,16^, which support longer first division times. This is related to the fact that, overall, the exponential model is not able to capture the observed cell dynamics for neither of the clonotypes, as can be seen in Fig. 7.

## Discussion

We analyse a multi-stage model of cell proliferation and death, tracking cell generations, in a framework that retains the benefits of a Markov process. With particular choices of rates, the models are equivalent to others in the literature^13,27,29,40–42^. In the case study of “Case study: lymphopenia-induced proliferation” section, the MS-G model performs better than the exponential model of time to division. The model implemented here provides a flexible framework for estimating the birth and death rates that describe the dynamics of lymphocyte populations^48,49^. The representation retains the advantages of a Markovian approach, including analytical tractability in some cases, and computational efficiency of numerical simulations with the Gillespie algorithm^50,51^. The expected number of cells in each generation satisfy a set of linear differential equations. Further comparison of this and of published models^13,27,29,40–42^ with different experimental datasets is the aim of future work.

It has been observed^13,15,16^ that immune cells typically need longer to divide for the first time, whereas later divisions require shorter times^5^. It is possible to assume that divided and undivided cells have different probability densities of time to cell division in exponential and Smith-Martin models^7,35,45^. With the multi-stage model introduced here, the separation need not be explicit because it is incorporated in the generation-dependent parameters. A longer mean time to first division, $\frac{N_0}{\lambda _0}$, than mean time to subsequent divisions, $\frac{N}{\lambda }$, is a natural part of the framework. Extension of the mathematical analysis in “Analytical results” section to the case $\lambda _0 \ne \lambda$, $N_0 \ne N$ and possibly generation-dependent death rate $\mu _g$, $g\ge 0$, would be desirable.

Our calculations rely on the assumption that cells are independent of each other. In particular, no fate correlation is assumed between daughter cells and their progenitors, or between siblings. However, data sets from time-lapse microscopy of B and T cell families^10–12,14,16,17,25^ show that division and death times for siblings are correlated, and “division destiny” is a familial characteristic^24^. A further potential extension of the MS-G model is the introduction of a population carrying capacity. In the model as described in “Analytical results” section, the mean number of cells over time either increases without bound, dies out or reaches a steady-state, depending on the relation between division-related parameters (birth rate and number of stages in the cell cycle), and the death rate. Competition for resources may be modelled using density-dependent birth and/or death rates^20,52^, or by rates that depend on the time-dependent availability of resources^53^.

## Data Availability

Python codes to carry out parameter calibration in the “Case study: lymphopenia-induced proliferation” section are available at https://doi.org/10.5281/zenodo.6337679.
